# Vancomycin associated acute kidney injury in patients with infectious endocarditis: a large retrospective cohort study

**DOI:** 10.3389/fphar.2023.1260802

**Published:** 2023-11-13

**Authors:** Pan Kunming, Huang Ying, Xu Chenqi, Chen Zhangzhang, Ding Xiaoqiang, Li Xiaoyu, Xu Xialian, Lv Qianzhou

**Affiliations:** ^1^ Department of Pharmacy, Zhongshan Hospital, Fudan University, Shanghai, China; ^2^ Department of Nephrology, Zhongshan Hospital, Fudan University, Shanghai, China; ^3^ Shanghai Key Laboratory of Kidney and Blood Purification, Shanghai Medical Center of Kidney Disease, Institute of Kidney Disease and Dialysis, Shanghai, China; ^4^ Department of Nephrology, Zhongshan Hospital, Fudan University, Xiamen, China

**Keywords:** vancomycin, acute kidney injury, infectious endocarditis, risk factors, duration of therapy

## Abstract

**Background:** Vancomycin remains the cornerstone antibiotic for the treatment of infective endocarditis (IE). Vancomycin has been associated with significant nephrotoxicity. However, vancomycin associated acute kidney injury (AKI) has not been evaluated in patients with IE. We conducted this large retrospective cohort study to reveal the incidence, risk factors, and prognosis of vancomycin-associated acute kidney injury (VA-AKI) in patients with IE.

**Methods:** Adult patients diagnosed with IE and receiving vancomycin were included. The primary outcome was VA-AKI.

**Results:** In total, 435 of the 600 patients were enrolled. Of these, 73.6% were male, and the median age was 52 years. The incidence of VA-AKI was 17.01% (74). Only 37.2% (162) of the patients received therapeutic monitoring of vancomycin, and 30 (18.5%) patients had reached the target vancomycin trough concentration. Multiple logistic regression analysis revealed that body mass index [odds ratio (OR) 1.088, 95% CI 1.004, 1.179], duration of vancomycin therapy (OR 1.030, 95% CI 1.003, 1.058), preexisting chronic kidney disease (OR 2.291, 95% CI 1.018, 5.516), admission to the intensive care unit (OR 2.291, 95% CI 1.289, 3.963) and concomitant radiocontrast agents (OR 2.085, 95% CI 1.093, 3.978) were independent risk factors for VA-AKI. Vancomycin variety (Lai Kexin vs. Wen Kexin, OR 0.498, 95% CI 0.281, 0.885) were determined to be an independent protective factor for VI-AKI. Receiver operator characteristic curve analysis revealed that duration of therapy longer than 10.75 days was associated with a significantly increased risk of VA-AKI (HR 1.927). Kidney function was fully or partially recovered in 73.0% (54) of patients with VA-AKI.

**Conclusion:** The incidence of VA-AKI in patients with IE was slightly higher than in general adult patients. Concomitant contrast agents were the most alarmingly nephrotoxic in patients with IE, adding a 2-fold risk of VA-AKI. In patients with IE, a course of vancomycin therapy longer than 10.75 days was associated with a significantly increased risk of AKI. Thus, closer monitoring of kidney function and vancomycin trough concentrations was recommended in patients with concurrent contrast or courses of vancomycin longer than 10.75 days.

## 1 Introduction

Vancomycin is a glycopeptide antibiotic that is active against Gram-positive bacteria since its approval in 1958 (Rybak et al., 2009; He et al., 2020; Rybak et al., 2020). Vancomycin remains the cornerstone antibiotic for the treatment of infective endocarditis (IE) and is the drug of first choice for *methicillin-resistant Staphylococcus aureus* (MRSA) infection (Chinese Society of Cardiology, 2014; Habib et al., 2015; Nakatani et al., 2019). The recommended duration of vancomycin therapy for IE is 4–6 weeks, with a target trough concentration of 15–20 mg/L, according to clinical guidelines for IE and guidelines for therapeutic monitoring of vancomycin (Rybak et al., 2009; Chinese Society of Cardiology, 2014; Habib et al., 2015; Nakatani et al., 2019; He et al., 2020; Rybak et al., 2020). However, vancomycin has been associated with significant nephrotoxicity. The incidence of vancomycin-associated acute kidney injury (VA-AKI) ranges from as low as 0% in the absence of concurrent risk factors to 43% (Filippone et al., 2017). Vancomycin is the second most common drug causing drug-induced hospital-acquired acute kidney injury (AKI) in China (Liu et al., 2021). Numerous risk factors have been defined for developing VA-AKI in patients receiving vancomycin, including maximal dose, duration of therapy, concomitant diseases, concomitant nephrotoxic drugs (Filippone et al., 2017; Jeffres, 2017; Kunming et al., 2021). Duration of vancomycin tended to be associated with an increased risk of AKI, and significantly positive durations include ≥7 days, ≥14 days and >15 days (Filippone et al., 2017; Kunming et al., 2021). The mechanism of vancomycin nephrotoxicity is dose dependent, thus high vancomycin target trough concentrations will increase the risk of AKI (Filippone et al., 2017; Jeffres, 2017). The recommended duration of vancomycin therapy for IE is 4–6 weeks, with a target trough concentration of 15–20 mg/L, which we hypothesized would result in an increased risk of VA-AKI in patients with IE. A small sample size retrospective study included 71 patients receiving vancomycin and showed that IE was significantly associated with an increased incidence of VA-AKI (OR = 7.63, 1.02–57.31) (Barberan et al., 2019).

IE is a rare infectious disease, but with high mortality and poor prognosis. The annual incidence ranges from 3 to 7 per 100,000 person-years in the most contemporary population surveys (Baddour et al., 2015). AKI is a common complication of IE and occurs in about 30% of patients (Chinese Society of Cardiology, 2014). Effective antimicrobial therapy such as vancomycin may reduce the complications of IE and the risk of progression to the need for surgery (Chinese Society of Cardiology, 2014; Baddour et al., 2015; Habib et al., 2015).

Gagneux-Brunon A. et al. described the frequency and risk factors for AKI during the course of IE in 112 patients and showed that vancomycin exposure was independently associated with AKI with an odds ratio of 1.084 (1.084–16.2) (Gagneux-Brunon et al., 2019). Similar results were obtained by Legrand M. et al. in their analysis of post-operative AKI following cardiac surgery for active IE, the use of vancomycin was found to be significantly associated with kidney function impairment (OR: 2.63, 2.07–3.34, *p* < 0.001) (Legrand et al., 2013). The results of most studies tended to support that vancomycin would increase the risk of AKI in patients with IE, however, vancomycin was not always a significant risk factor for AKI in patients with IE (Goenaga Sanchez et al., 2017; Ritchie et al., 2017). Ritchie, B. M., et al. evaluated AKI in 211 patients with IE, and multivariate analysis showed that vancomycin combined with aminoglycosides was significantly associated with an increased risk of kidney failure in patients with bacterial endocarditis, whereas vancomycin alone did not significantly increase the risk (Ritchie et al., 2017). Whether vancomycin increases the risk of AKI in patients with IE remains a minor controversy.

Vancomycin is associated with significant nephrotoxicity, and the risk will be further increased by the long course of treatment and high trough concentrations. Meanwhile, AKI is one of the common complications of IE. Therefore, the risk of AKI in patients with IE who are treated with vancomycin would be of great concern. VA-AKI is associated with prolonged hospital stays, the need for additional antibiotic therapy, and, in rare cases, dialysis treatment, as well as increased medical costs and mortality (Jeffres, 2017). Based on a comprehensive literature search, there are no published studies assessing the characteristics of VA-AKI in this specific group of patients with IE. We conducted this large retrospective cohort study to reveal the incidence, risk factors, and prognosis of VA-AKI in patients with IE in order to provide a clinical reference for the prevention and reduction of VA-AKI in patients with IE.

## 2 Methods

### 2.1 Study design and patients

This was a retrospective observational cohort study conducted at Zhongshan Hospital of Fudan University, a 2005-bed top-tier general teaching hospital in China. This study was reviewed and approved by the Ethics Committee of Zhongshan Hospital, Fudan University (Shanghai, China, approval number: B2019-194 (2)), and no consent was needed. The study is reported according to Strengthening The Reporting of Observational Studies in Epidemiology (STROBE) guidelines (von Elm et al., 2007). All consecutive adult (≥18 years) patients admitted between January 2016 and June 2019 with a diagnosis of IE according to the modified Duke criteria (Chinese Society of Cardiology, 2014; Baddour et al., 2015; Habib et al., 2015) and received vancomycin anti-infective therapy were included. Patients were excluded if: 1) they had chronic kidney disease (CKD) stage 5 or were on regular dialysis. 2) their baseline serum creatinine (SCr) was ≥4 mg/dL (353.6 μmol/L). 3) they had AKI at admission. 4) they had a history of nephrectomy, kidney transplantation, or sole kidney. 5) their vancomycin administration was not intravenous. 6) they received less than 4 doses of vancomycin. 7) their SCr measurement was insufficient to determine whether AKI had developed (SCr was not measured within 7 days before receiving vancomycin, or SCr was not measured within 7 days after stopping vancomycin, or SCr was not measured within 7 days before receiving and after stopping vancomycin, or SCr was measured before receiving and after stopping vancomycin, but the interval was longer than 7 days, so as not be able to assess the occurrence of acute kidney injury).

### 2.2 Data collection

Data were collected from the hospital’s electronic database between December 2021 and November 2022 using a standardized case report form. Patient information was anonymized by a researcher not involved in the study. Patients were not followed up after discharge. The following variables were collected: demographic information, concomitant underlying diseases, severity of disease, vancomycin exposure, vancomycin variety (Laikexin vs. Wenkexin; trade name: Laikexin, generic name: Vancomycin Hydrochloride for Injection, manufacturers: Zhejiang Medicine Co., Ltd. Xinchang Pharmaceutical Factory, China, specification: 500 mg/bottle; trade name: Wenkexin, generic name: Vancomycin HydrochlorPide for Injection, manufacturer: VIANEX S.A. (PLANT C), Greece, specification: 500 mg/bottle), therapeutic drug monitoring (TDM) during hospitalization, and concomitant nephrotoxic drugs. The detailed items of data collection are presented in Table 1. Missing values were filled using the median. We used the 2012 Kidney Disease: Improving Global Outcomes (KDIGO) Clinical practice guideline for AKI to define and stage AKI, e.g., an increase in SCr by ≥ 0.3 mg/dL (≥26.5 μmol/L) within 48 h or an increase in SCr to ≥1.5 times baseline, which was known or presumed to have occurred within the prior 7 days (Khwaja A., 2012). AKI severity was described by the highest stage of AKI (1, 2, or 3) and receipt of renal replacement therapy (RRT), according to the KDIGO criterion. Preexisting CKD is defined as an estimated glomerular filtration rate (eGFR) of less than 60 mL/(min·1.73 m^2^) (Calculated by the formula of the Chronic Kidney Disease Epidemiology Collaboration equation, CKD-EPI). TDM is prescribed by clinicians based on their own experience. Nephrotoxic drugs were documented as loop diuretics, aminoglycosides, cephalosporins, carbapenems, renin-angiotensin system blockers, radiocontrast agents and non-steroidal anti-inflammatory drugs.

**TABLE 1:** Demographic information, clinical characteristics, medication exposure of patients with and without VA-AKI.

Factors	Total N = 435	Patients without VA-AKI N = 361	Patients with VA-AKI N = 74	*p*-value
*Demographic information*				
Gender (male)	320 (73.6)	267 (74.0)	54 (71.6)	0.68
Age (years)	52.0 (22)	51.0 (22.0)	58.0 (22.0)	0.03
Body Mass Index (kg/m^2^)^a^	21.8 (4.2)	21.48 (4.6)	22.17 (4.13)	0.18
Payment mode				0.13
At one’s own expense	272 (62.5)	220 (60.9)	52 (70.3)	
National basic medical insurance	163 (37.5)	141 (39.1)	22 (29.7)	
*Concomitant underlying diseases*				
Preexisting chronic kidney disease	37 (8.5)	10 (13.5)	27 (7.5)	0.09
Coronary heart disease	23 (5.3)	20 (5.5)	3 (4.1)	0.81^b^
Hypertension	66 (15.2)	53 (14.7)	13 (17.6)	0.53
Diabetes	28 (6.4)	21 (5.8)	7 (9.5)	0.37^b^
Heart failure	51 (11.7)	40 (11.1)	11 (14.9)	0.36
Sepsis	144 (33.1)	119 (33)	25 (33.8)	0.89
Cancer	43 (9.9)	36 (10)	7 (9.5)	0.89
valvular heart disease	378 (86.9)	311 (86.1)	67 (90.5)	0.31
*Severity of illness*				
Baseline serum creatinine μmol/L	77 (30.0)	76.0 (29.9)	86.3 (41.0)	0.01
Cardiac surgery	365 (83.9)	297 (82.3)	68 (91.9)	0.04
Admission to the ICU	220 (50.6)	175 (48.5)	45 (60.8)	0.053
Mechanical ventilation	67 (15.4)	53 (14.7)	14 (18.9)	0.36
*Vancomycin exposure*				
Vancomycin variety				0.002
Lai Kexin	300 (69.0)	238 (65.9)	62 (83.8)	
Wen Kexin	135 (31.0)	123 (34.1)	12 (16.2)	
Duration of vancomycin therapy mean (median), range	10.0 (9.0), 1.5–56.0	9.5 (8.5), 1.5–49.5	12.0 (10.6), 1.5–56.5	0.04
Daily dose				0.11
≤2 g/d	389 (89.4)	319 (88.4)	70 (94.6)	
>2 g/d	46 (10.6)	42 (11.6)	4 (5.4)	
Trough concentration >15 mg/L^a^	95 (57.4)	63 (52.7)	30 (73.2)	0.018
*Concomitant nephrotoxic drugs*	370 (85.1)	303 (83.9)	67 (90.5)	0.15
Loop diuretics	350 (80.5)	287 (79.5)	63 (85.1)	0.27
Aminoglycosides	11 (2.5)	10 (2.8)	1 (1.4)	0.76^b^
Cephalosporins	81 (18.6)	63 (17.5)	18 (24.3)	0.17
Carbapenems	203 (46.7)	163 (45.2)	40 (54.1)	0.16
RAS blockers	45 (10.3)	35 (9.7)	10 (13.5)	0.33^b^
Radiocontrast agents	62 (14.3)	45 (12.5)	17 (23.0)	0.02
NSAIDs	25 (5.7)	21 (5.8)	4 (5.4)	1.00^b^
*Concomitant nephroprotective drugs*				
Vasopressors	103 (23.7)	81 (22.4)	22 (29.7)	0.18
Glutathione	69 (15.9)	56 (15.5)	13 (17.6)	0.66
Coenzyme Q10	29 (6.7)	20 (5.5)	9 (12.2)	0.07^b^

Data are described as mean (SD), n (%), or median (IQR).

^a^ There were missing values in the BMI, data. The percentage of missing values for total was 8.8% (8.9%for patients without VA-AKI; 8.1% for patients with VA-AKI). Missing values were filled using the median. *There were missing values in the date of trough concentration >15 mg/L. The percentage of missing values for total was 62.8% (273): 66.5% (240) for patients without VA-AKI; 44.6% (33) for patients with VA-AKI.

^b^ Refers to the calibration of the chi-square test. ICU, intensive care unit; NSAIDs , Non-steroidal anti-inflammatory drugs. VA-AKI , vancomycin-associated kidney injury. RAS, blockers = Renin-angiotensin system blockers. Chronic kidney disease is defined as an estimated glomerular filtration rate (eGFR) of less than 60 mL/(min·1.73 m2) (Calculated by the formula of the Chronic Kidney Disease Epidemiology Collaboration equation, CKD-EPI).

### 2.3 Outcome measure

The primary outcome measure was VA-AKI, defined as patients who developed AKI during vancomycin therapy or within 48 h of vancomycin discontinuation. For patients who developed AKI, we further evaluated the 30-day morbidity and recovery of kidney function. Kidney recovery was categorized into three levels: full recovery, partial recovery, and failure to recover. We defined full recovery as a decrease in SCr to baseline at discharge. We defined partial recovery as a decrease in SCr of 25% or more from the peak concentration, but still above baseline. We defined failure to recover as the patient remaining dependent on dialysis or SCr decreasing by less than 25% from peak concentration until discharge (Yang et al., 2015; Pan et al., 2017; Pan et al., 2018).

### 2.4 Data analysis

We used the Kolmogorov-Smirnov test to assess the normality of the variables. Continuous variables were presented as means with standard deviations or medians with interquartile ranges (IQR), and we used independent t-tests or rank-sum tests to compare variables between groups. Qualitative variables are presented as frequencies with corresponding percentages, and we used chi-squared or Fisher’s exact tests to compare variables between groups.

We used multivariate logistic regression analysis to assess independent risk factors for the development of VA-AKI, as well as the non-recovery of kidney function. We included all covariates with a *p*-value ≤ 0.05 in univariate analysis and forced other relevant variables into multivariable models. A backward stepwise regression was used to construct the final model. The following covariates were included in the model to explore the risk factors for the occurrence of VA-AKI: gender (male vs. female), age (years), body mass index (BMI), preexisting CKD, cardiac surgery (yes or no), admission to the ICU (yes or no), vancomycin variety (Lai Kexin vs. Wen Kexin), length of vancomycin therapy (days), vancomycin daily dose (≤2 g/d vs. > 2 g/d), concomitant nephrotoxic drugs (yes or no), concomitant radiocontrast agents (yes or no). The results of the univariate analysis of factors affecting the recovery of kidney function in patients with VA-AKI were shown in Supplementary Table S1. The following covariates were included in the model to explore the risk factors for non-recovery of kidney function in patients with VA-AKI: age (years), payment mode (At one’s own expense vs. National basic medical insurance), concomitant vasopressors (yes or no), and concomitant carbapenems (yes or no). The good of fit was evaluated by the analysis of Hosmer and Lemeshow. Cut-off values for vancomycin trough concentrations and duration of therapy that contributed to the development of VA-AKI were derived by receiver operating characteristic curve (ROC) analysis. All *p*-values were two-sided, and a *p*-value ≤ 0.05 was considered statistically significant. All statistical analyses were performed using SPSS statistics version 26.0 (IBM Inc., Armonk, NY, United States).

## 3 Results

### 3.1 Patients’ characteristics

**FIGURE 1:**
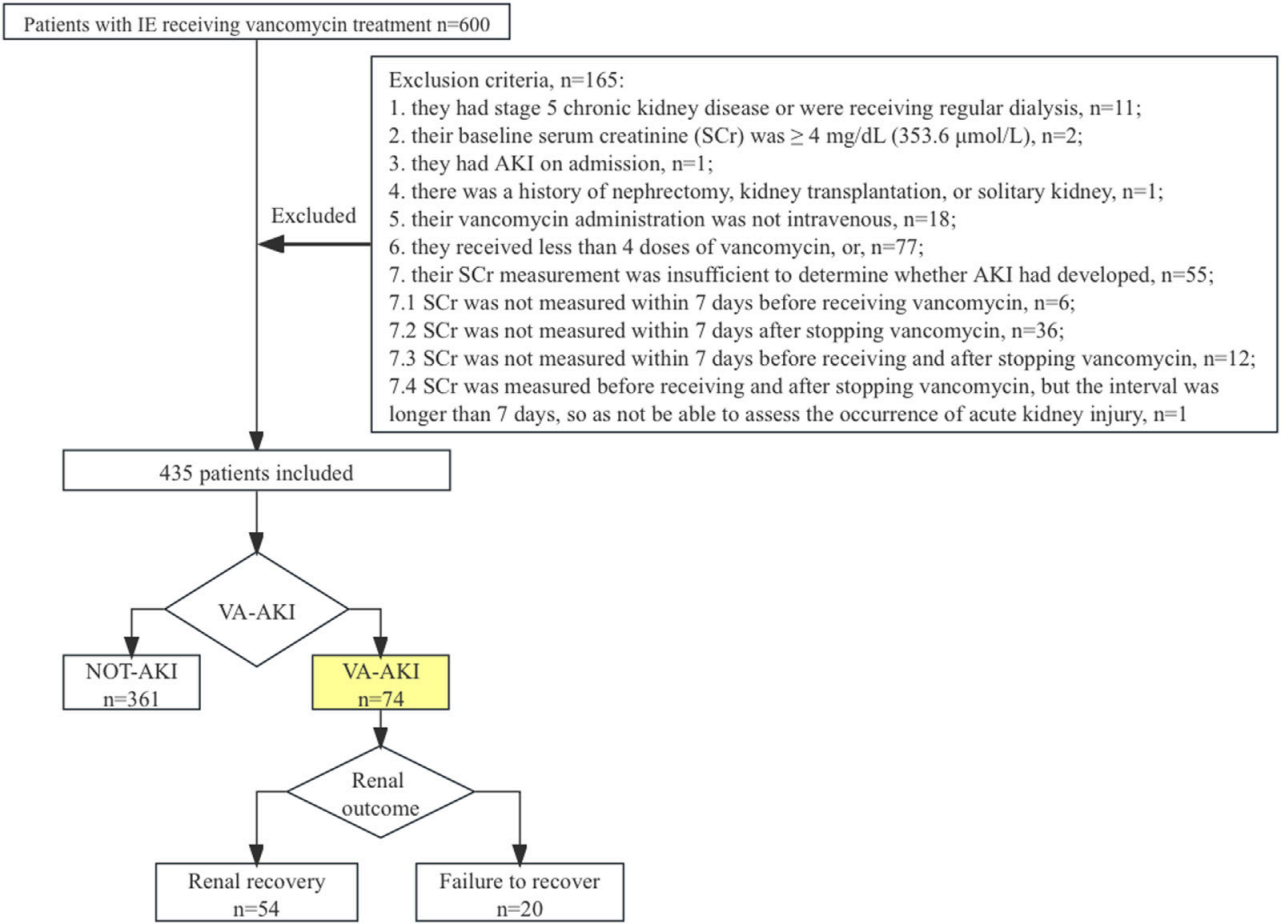
Study design.

There were 600 patients evaluated for study inclusion. After applying the exclusion criteria, 165 (27.5%) patients were omitted from the study. Of those excluded, 55 patients lacked SCr measurements, typically within 7 days after stopping vancomycin therapy (Figure 1). In total, 435 patients were included for analysis. Of these, 73.6% were male, and the median age was 52 (IQR = 22) years. A total of 40 patients (8.8%) received heart valve surgery during hospitalization. The remaining patients were treated with vancomycin-based anti-infective therapy only.

Seventy-four patients developed AKI. The incidence of VA-AKI was 17.0% (74), and the time of diagnosis was day 5.9 (IQR = 7.4) after receiving vancomycin therapy. Table 1 displayed patient demographic information, concomitant underlying diseases, and severity of illness. Patients with AKI tend to be older (58.0 vs. 51.0 years, *p* = 0.028) and were more likely to concomitant preexisting CKD (13.5%vs. 7.5%, *p* = 0.09) and to undergo cardiac surgery (91.9% vs. 82.3%, *p* = 0.040), compared to patients without AKI. Table 1 also listed patient vancomycin exposure and concomitant nephrotoxic drugs. There were two varieties of vancomycin from different companies. Patients with VA-AKI were more likely to receive Lai Kexin (Lai Kexin vs. Wen Kexin 83.8% vs. 65.9%, *p* = 0.002), compared with patients without VA-AKI. Patients with AKI underwent a longer duration of vancomycin treatment (12.0 ± 10.6 days vs. 9.5 ± 8.5 days, *p* = 0.038), higher proportions of vancomycin concentrations greater than 15 mg/L (73.2% vs. 52.7, *p* = 0.018) and were more likely to concomitant radiocontrast agents (loversol, iopromid) (23.0% vs. 12.5%, *p* = 0.019). Twenty-three patients (5.3%) received vancomycin for more than 4 weeks.

### 3.2 Therapeutic drug monitoring of patients

One hundred and sixty-two (37.2%) patients received TDM during vancomycin therapy. Of the patients who received TDM, 8% (13) had their initial vancomycin TDM on day 3, in accordance with the guideline recommendations, and 88.3% (143) of patients had delayed first vancomycin trough concentration monitoring. Only 30 (18.5%) patients had vancomycin trough concentrations between 15–20 mg/L and reached the target trough concentration. Besides, 69 (42.6%) patients had vancomycin trough concentrations <15 mg/L and 63 (38.9%) patients had vancomycin trough concentrations >20 mg/L. The incidence of VA-AKI in patients receiving TDM was 25.3% (41) and the risk of VA-AKI increased with higher vancomycin trough concentrations. The VA-AKI rates were 17.3% (4), 15.2% (7), 23.3% (7), and 36.3% (23) for trough concentrations of <10 mg/L, ≥10 - <15 mg/L, ≥15 - ≤20 mg/L, and >20 mg/L, respectively (*p* < 0.058). By binary logistic analysis, the odds ratios (OR) were 0.853 (*p* = 0.82), 1.446 (*p* = 0.60), and 2.731 (*p* = 0.099) for the development of VA-AKI at trough concentrations ≥10 - <15 mg/L, ≥15 - ≤20 mg/L, and >20 mg/L, respectively, compared to trough concentrations <10 mg/L. ROC analysis revealed a cut-off value of 25.5 mg/L for trough concentrations that contributed to the development of VA-AKI (AUC = 0.654, 95% CI 0.557, 0.751, *p* = 0.0032).

### 3.3 Comparison of medical costs and outcomes for patients with and without VA-AKI

Patients who developed AKI had longer hospital stays (18.0 vs. 15.1 days, *p* = 0.005) and a higher 30-day mortality rate (4.4% vs. 0.6%, *p* = 0.037) than those who did not develop AKI. Patients with VA-AKI were more likely to have higher total costs (119.8 vs. 98.4 thousand US dollars, *p* = 0.001), medical costs (*p* < 0.001), treatment costs (*p* < 0.001), and medical consumables costs (*p* = 0.011), compared with patients without VA-AKI (Table 2).

**TABLE 2:** Medical costs and outcomes of patients with and without vancomycin-associated acute kidney injury.

	Patients without va-AKI N = 361	Patients with va-AKI N = 74	*p*-value
Length of hospital stay (day)	15.1 (10.1)	18.0 (12.0)	0.005
Need for Dialysis	0 0)	5 (5.4)	<0.001
30-day mortality	2 (0.6)	3 (4.1)	0.037^f^
Total costs (thousand US$)	98.4 (61.1)	119.8 (79.3)	0.001
Medicine costs (thousand US$)	20.7 (13.7)	29.4 (17.6)	<0.001
Treatment costs (thousand US$)	2.9 (2.1)	3.6 (2.8)	<0.001
medical consumables costs (thousand US$)	51.9 (52.0)	61.7 (49.4)	0.011

Data are described as mean (SD), n (%), or median (IQR). VA-AKI, vancomycin-associated kidney injury. F refers to Fisher’s exact test.

### 3.4 Risk factors for VA-AKI

**FIGURE 2:**
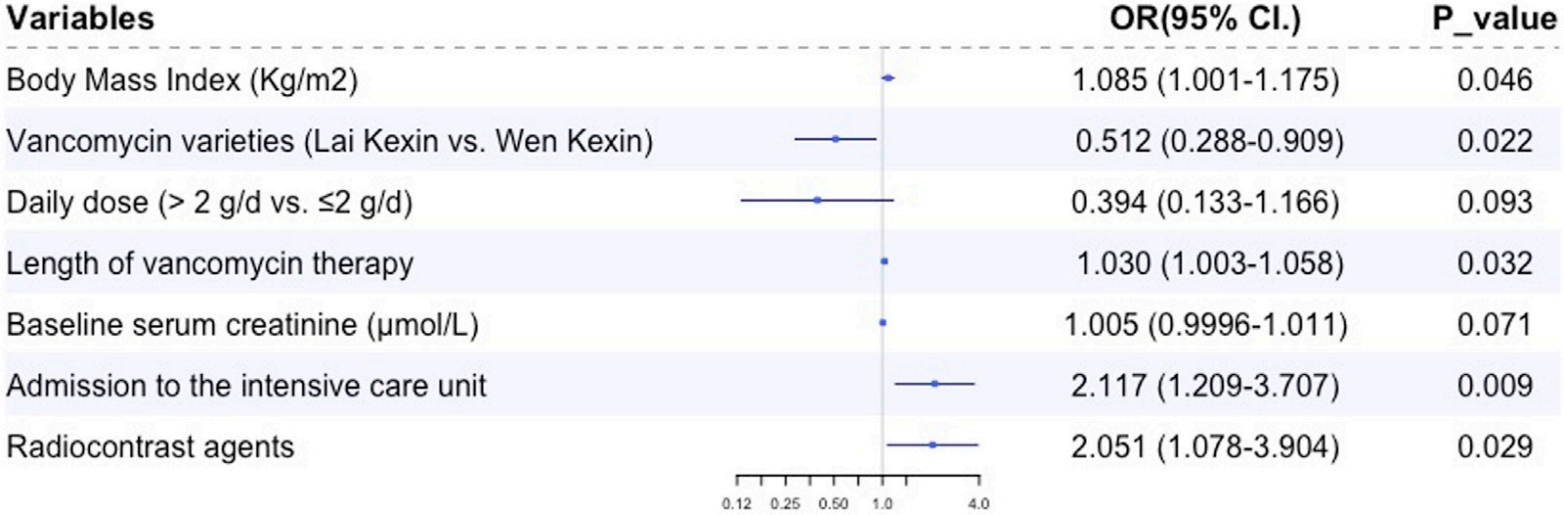
Forest-plot figure of risk factors for vancomycin-associated acute kidney injury.

Multiple logistic regression analysis revealed that BMI (OR 1.085, 95% CI 1.004, 1.179, *p* = 0.039), duration of vancomycin therapy (OR 1.030, 95% CI 1.003, 1.058, *p* = 0.032), preexisting CKD (OR 2.291, 95% CI 1.018, 5.516, *p* = 0.045), admission to the ICU (OR 2.260, 95% CI 1.289, 3.963, *p* = 0.004) and concomitant radiocontrast agents (OR 2.085, 95% CI 1.093, 3.978, *p* = 0.029) were independent risk factors for VA-AKI; vancomycin variety (Lai Kexin vs. Wen Kexin, OR 0.498, 95% CI 0.281, 0.885, *p* = 0.017) were determined to be an independent protective factor for VI-AKI (Table 3; Figure 2). ROC analysis revealed a cut-off value of 10.75 days for the duration of vancomycin therapy that contributed to the development of VA-AKI. Univariate analysis revealed a significantly higher risk of VA- AKI in patients with a long duration of therapy (longer than 10.75 days) compared to those with a short duration of therapy (shorter than 10.75 days) (HR 1.927, 95% CI 1.159 3.205, *p* = 0.011).

**TABLE 3:** Risk factors for vancomycin-associated acute kidney injury.

	B	S.E.	OR	95% CI. For OR		*p*-value
				Lower	Upper	
Body Mass Index (kg/m^2^)	0.0840	0.041	1.088	1.004	1.179	0.039
Vancomycin varieties (Lai Kexin vs. Wen Kexin)	−0.696	0.293	0.498	0.281	0.885	0.017
Daily dose (>2 g/d vs. ≤2 g/d)	−0.911	0.554	0.402	0.136	1.190	0.010
Length of vancomycin therapy	0.030	0.014	1.030	1.003	1.058	0.031
Preexisting chronic kidney disease	0.829	0.414	2.291	1.018	5.516	0.045
Admission to the intensive care unit	0.815	0.287	2.260	1.289	3.963	0.004
Radiocontrast agents	0.735	0.330	2.085	1.093	3.978	0.026
Constant	−4.136	0.987	0.016			<0.001

### 3.5 Treatment and outcome of patients with vancomycin-associated acute kidney injury

The 30-day mortality rate in patients with VA-AKI was 4.1% (3). 5.4% (4) of patients were treated with dialysis. Kidney function was fully or partially recovered in 73.0% (54) of patients, and 27.0% (20) failed to recover kidney function until discharge (Table 4). Multiple logistic regression analysis showed payment mode (at one’s own expense vs. national basic medical insurance) was the only independent risk factor for non-recovery of kidney function in patients with VA-AKI (OR 4.78, 95% CI 1.59, 14.38, *p* = 0.005).

**TABLE 4:** Treatment and outcome of patients with vancomycin-associated acute kidney injury.

	Total N = 74	Stage 1 N = 55	Stage 2 N = 12	Stage 3 N = 7	*p*-Value
30-day mortality n (%)	3 (4.1)	1 (1.8)	1 (8.3)	1 (14.3)	0.21
Receive dialysis n (%)	4 (5.4)	1 (1.8)	1 (8.3)	2 (28.6)	0.011
Kidney recovery n (%)	54 (73.0)	40 (72.7)	10 (83.3)	4 (57.1)	0.46^a^
Failure to recover n (%)	20 (27.0)	15 (27.3)	2 (16.7)	3 (42.9)	
Full recovery n (%)	32 (43.2)	27 (49.1)	3 (25.0)	2 (28.6)	—
Partial recovery n (%)	22 (29.7)	13 (23.6)	7 (58.3)	2 (28.6)	—

^a^ Kidney recovery group vs. Failure to recover group.

## 4 Discussion

**FIGURE 3:**
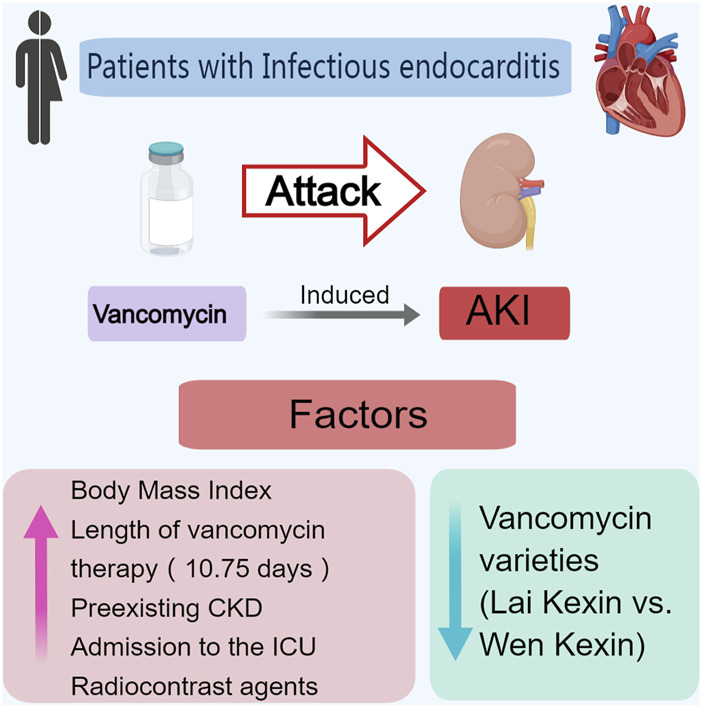
Graphical abstract.

This study demonstrated the incidence of VA-AKI in patients with IE, which was 17.0%, and further revealed the clinical characteristics, risk factors, and outcomes of VA-AKI. To the best of our knowledge, this is the first study to thematically investigate VA-AKI in patients with IE, and we believe that the results will provide an important reference for the rational use of vancomycin in this special population (Figure 3).

Several studies have focused on the nephrotoxicity of antimicrobial agents used to treat IE, the majority focusing on aminoglycosides such as gentamicin-associated nephrotoxicity (Cosgrove et al., 2009). However, physicians still did not pay enough attention to VA-AKI in patients with IE. The incidence of IE is low, and the sample sizes of published studies are relatively small, in the range of 100–200 cases (Legrand et al., 2013; Ritchie et al., 2017; Barberan et al., 2019). This study included 435 patients with, IE who were treated with vancomycin, which, to our knowledge, is the largest sample size of any study investigating antibiotic-associated AKI in patients with IE. In previous studies involving vancomycin-associated kidney injury, vancomycin was generally used as conventional therapy or as a control, compared with daptomycin (Barberan et al., 2019). Studies showed vancomycin to be less nephrotoxic than daptomycin, but greater than or closer to other antibiotics such as beta-lactam antibiotics (Barberan et al., 2019; Blevins et al., 2019; Muklewicz et al., 2021). However, no studies have evaluated the incidence, clinical characteristics, risk factors, and outcomes of VA-AKI in patients with IE.

This study showed a 17.0% prevalence of VA-AKI in adult patients with IE, which is slightly higher than in the general adult population, who are not a special population such as obese, critically ill, or elderly (Pan et al., 2018; Chun et al., 2021; Contejean et al., 2021; Kunming et al., 2021; Muklewicz et al., 2021; Kiley et al., 2022). Data from studies in the United States (11.4%) (Muklewicz et al., 2021), Chinese mainland (14.3%) (Kunming et al., 2021), Taiwan in China (11.5%) (Kiley et al., 2022), South Korea (15.38) (Chun et al., 2021), France (11.2%) (Contejean et al., 2021) and other countries or regions indicated that the incidence of VA-AKI in adult patients was generally around 10%–15%. However, the incidence of VA-AKI in patients with IE is lower than in obese patients and critically ill patients (Filippone et al., 2017). We speculate that there are four causes for the higher incidence of VA-AKI in patients with IE than in the general adult population. First, pathogenic bacteria that infect the endocardium may also invade the kidney and induce AKI through mechanisms such as immune complexes and vasculitis glomerulonephritis (Habib et al., 2015; Ritchie et al., 2017; Gagneux-Brunon et al., 2019). Second, there is a relatively high rate of patients with IE undergoing cardiac surgery, especially valve surgery (Legrand et al., 2013; Gagneux-Brunon et al., 2019). Third, the course of vancomycin for IE is long, usually 4–6 weeks (Chinese Society of Cardiology, 2014; Baddour et al., 2015; Habib et al., 2015). Fourth, a high percentage of patients with IE received vancomycin concomitant contrast agents (Legrand et al., 2013; Mehran et al., 2019). Moreover, these possible contributing factors will also provide an indication for reducing the risk of VA-AKI in patients with IE.

The study showed that 37.2% of patients with IE received vancomycin TDM, 8% of patients were initially monitored on day 3 of vancomycin therapy, and 18.5% of patients achieved target trough concentrations. According to the vancomycin TDM guidelines published by the American Society of Health-System Pharmacists in 2009 and the Chinese Pharmacological Society in 2020 (Rybak et al., 2009; He et al., 2020), there were three deficiencies in vancomycin TDM: low vancomycin TDM rate, inaccurate timing of initial TDM, and low attainment of target trough concentrations. Vancomycin concentration monitoring in most hospitals in China, as exemplified by our hospital, is mainly based on the experience and expertise of clinicians. The technology for TDM of vancomycin is well established and the guidelines are clear in the recommendations for the management of vancomycin monitoring, however, the status of monitoring in clinical practice is not satisfactory. We previously conducted a meta-analysis including 19 studies with 2,598 patients from developed countries such as the United States, Japan, Australia, and New Zealand, and developing countries such as China and India, and showed that the mean vancomycin trough concentration attainment rate was 34.3% under the conventional physician-led model of care (Kunming et al., 2023). Factors contributing to the unsatisfactory status of monitoring under physician-led vancomycin TDM included negative perception towards prescription guidelines, lack of knowledge regarding TDM guidelines, the hierarchy of medication management, work pressure, and ineffective communication among healthcare providers (Abdel Jalil et al., 2023). Our further analysis showed that 64.6% (281) of the patients were given vancomycin at 1g q12 h, which was a conventional dose. Patients with IE may have complex pathophysiological profiles and vancomycin pharmacokinetics; therefore, more precise dosing regimens, more frequent concentration monitoring, and dose adjustments are necessary (Elyasi et al., 2012; Filippone et al., 2017; Rybak et al., 2020). We also found that the hospitalization units for patients with IE were predominantly surgical. Surgeons may be more focused on surgical treatment and pay less attention to drug therapy. The 2020 update of the vancomycin TDM guidelines further recommended area under the curve (AUC)-guided dosing and monitoring regimens, which raised the difficulty of vancomycin monitoring (Rybak et al., 2020). Considering that some medical institutions do not have the technology and professional staff to monitor AUC, and the feasibility of monitoring trough concentration is better than monitoring AUC, thus the evidence-based guideline for therapeutic drug monitoring of vancomycin: 2020 update by the division of therapeutic drug monitoring, Chinese pharmacological society, recommends monitoring trough concentration or AUC (He et al., 2020). We believe that this study reveals the deficiencies in vancomycin prescribing and TDM and suggests that clinicians should pay attention to these issues. In addition, we suggest clinical care teams should increase the involvement of clinical pharmacists. Our previously published study showed that pharmacist intervention in vancomycin treatment significantly decreased the rate of VA-AKI while improving efficacy and reducing mortality (Kunming et al., 2023). Clinical pharmacists are experts in medication therapy management, and many hospitals, mainly in the United States, have established the model of pharmacist managing vancomycin dosing and monitoring, which is worthy of emulation by hospitals in other countries (Kunming et al., 2023).

Concomitant nephrotoxic drugs were risk factors for VA-AKI, especially aminoglycosides and piperacillin-tazobactam (Kim JY et al., 2022; Filippone et al., 2017; O'Callaghan et al., 2020). According to this study, concomitant contrast media was the most alarmingly nephrotoxic agent in patients with IE. Concomitant contrast resulted in a 2.085-fold increased risk of VA-AKI. Contrast-associated AKI was one of the leading causes of iatrogenic kidney insufficiency (Huang et al., 2022). Direct mechanisms of contrast-associated AKI were due to nephrotoxic effects on the tubular epithelium, leading to loss of function, apoptosis, and eventually, necrosis. In addition, contrast can induce ischemic injury through an indirect mechanism of regional or global perfusion reduction (Mehran et al., 2019). The mechanism of vancomycin-associated AKI was suggested to be associated with the oxidative stress effect on proximal kidney tubular (Elyasi et al., 2012; Jeffres, 2017). Contrast and vancomycin have a synergistic effect on the mechanism of kidney injury; therefore, patients with IE require special attention to the synergistic risk of vancomycin concurrent with contrast. We recommend to postpone tests requiring contrast, such as coronary angiograms and coronary artery imaging, during vancomycin therapy, if conditions permit; or to replace vancomycin with teicoplanin.

The duration of vancomycin therapy was a factor that we focused on. The recommended course of vancomycin for the medical treatment of patients with IE was 4–6 weeks, which was longer than the course of most infections (Chinese Society of Cardiology, 2014; Baddour et al., 2015; Habib et al., 2015). This study suggested that duration of therapy was an independent risk factor for VA-AKI in patients with IE, consistent with previous studies (Jeffres, 2017). However, the HR for duration of vancomycin therapy was only 1.030, showing a weak increased risk of AKI, which was lower than we expected. The risk of developing vancomycin nephrotoxicity significantly increased with a duration ≥7 days, and 14 days (Gagneux-Brunon et al., 2019; Mehran et al., 2019; Rybak et al., 2020). The course of vancomycin in this study was 12 and 9.5 days for patients who developed and did not develop AKI, both greater than 7 and less than 14 days, which may account for the low HR for the vancomycin course. Univariate analysis showed a significantly higher risk of AKI in patients with a long duration of treatment (longer than 10.75 days) compared to those with a short duration of treatment (shorter than 10.75 days) (HR 1.927, *p* = 0.011). In patients with IE, we suggested that the course of vancomycin longer than 10.75 days was associated with a significantly increased risk of AKI and required more frequent monitoring of kidney function and vancomycin trough concentrations.

This study showed that BMI and residence in the ICU were risk factors for VA-AKI in patients with IE, which was consistent with the findings in the general adult patients (Liu et al., 2021; Kim JY et al., 2022). Several studies have shown that obesity or increasing BMI was significantly associated with the development of VA-AKI (Choi et al., 2017). Obese patients have a significantly higher incidence of trough levels >20 mg/L due to complex vancomycin pharmacokinetics (e.g., increased volume of distribution and clearance) and demanding dose adjustment regimens (Elyasi et al., 2012). In addition, a study by Maha S. Assadoon et al. found that obese patients may experience vancomycin accumulation within the first 10 days of treatment (Assadoon et al., 2022). Measures such as actual weight-based dosing, frequent monitoring of vancomycin, and AUC-guided therapy may contribute to the reduction of VA-AKI in obese patients (Choi et al., 2017; Rybak et al., 2020; Assadoon et al., 2022). Severity of illness impacted development of AKI in patients receiving vancomycin. Admission to the ICU indicated that patients were critically ill and more likely to have a combination of multiple risk factors for AKI, thus increasing the nephrotoxicity of vancomycin (Filippone et al., 2017; Blevins et al., 2019; O'Callaghan et al., 2020).

VA-AKI was associated with increased length of hospital stay, need for dialysis, increased mortality, and increased costs, which is consistent with previous studies (Jeffres, 2017). More than 70% of the patient’s kidney function can be recovered, which suggests the importance of early intervention and aggressive salvage of VA-AKI. Payment method (at one’s own expense vs. national basic medical insurance) was the only risk factor for kidney function recovery, and this was not reported in previous studies. We speculate that patients without medical insurance may limit the choice of measures for improving kidney function due to their weak payment willingness.

This study has several strengths. First, the sample size of this study was relatively large and was the largest sample size studying antibiotic-associated kidney injury in patients with IE. Second, we counted the consequences of VA-AKI, including economic consequences, mortality, and recovery of kidney function. We further analyzed the risk factors affecting the recovery of kidney function in patients with AKI. Third, the economic consequences of VA-AKI were rarely addressed in previous studies. However, the study has several limitations. First, this was a single-center retrospective study, and we could only demonstrate a correlation between vancomycin and AKI, not a causal relationship. Second, vancomycin trough concentrations were not included as a risk factor due to the low percentage of TDM and the small amount of concentration data available for analysis. For the available data, we analyzed the correlation between vancomycin trough concentrations and the risk of AKI occurrence. However, we believe that this deficiency does not affect the quality of this study. Globally, especially in developing countries, hospitals without vancomycin concentration monitoring technology remain in the majority. Vancomycin dose and duration are important bases for assessing vancomycin exposure, and both variables were included in this study. Moreover, even in hospitals where vancomycin monitoring is carried out, its actual monitoring is not satisfactory. Third, this study did not collect data on urine volume, which is an important criterion for the diagnosis of AKI. Due to the retrospective nature of this study, medical record data on urine volume were not or inaccurately recorded, and we will address this shortcoming in a prospective study to be conducted in the future. Fourth, we did not follow up with the patient after discharge. Because the patient’s information was anonymized and their contact information was not available.

## 5 Conclusion

The incidence of VA-AKI in patients with IE was slightly higher than in general adult patients, and lower than in special populations such as obese and critically ill. Concomitant contrast agents were the most alarmingly nephrotoxic in patients with IE, adding a 2-fold risk of VA-AKI, and we speculate that this may be due to a synergistic mechanism of nephrotoxicity between contrast agents and vancomycin. In patients with IE, a course of vancomycin therapy longer than 10.75 days was associated with a significantly increased risk of AKI and required more frequent monitoring of kidney function and vancomycin trough concentrations.

## Data Availability

The raw data supporting the conclusion of this article will be made available by the authors, without undue reservation.

## References

[B1] Abdel Jalil M. H. Etaijazeen R. Khaled Abu-Mahfouz F. Abu Hammour K. Hasan Matalqah M. Saleh Khaleel Albadaineh J. (2023). Vancomycin prescribing and therapeutic drug monitoring: challenges of real clinical practice. PLoS One 18 (5), e0285717. 10.1371/journal.pone.0285717 37195936 PMC10191297

[B2] Assadoon M. S. Pearson J. C. Kubiak D. W. Kovacevic M. P. Dionne B. W. (2022). Evaluation of vancomycin accumulation in patients with obesity. Open Forum Infect. Dis. 9 (10), ofac491. 10.1093/ofid/ofac491 36267260 PMC9578159

[B3] Baddour L. M. Wilson W. R. Bayer A. S. Fowler V. G. Jr Tleyjeh I. M. Rybak M. J. (2015). Infective endocarditis in adults: diagnosis, antimicrobial therapy, and management of complications: a scientific statement for healthcare professionals from the American heart association. Circulation 132 (15), 1435–1486. 10.1161/CIR.0000000000000296 26373316

[B4] Barberan J. Mensa J. Artero A. Epelde F. Rodriguez J. C. Ruiz-Morales J. (2019). Factors associated with development of nephrotoxicity in patients treated with vancomycin versus daptomycin for severe Gram-positive infections: a practice-based study. Rev. espanola Quimioter. 32 (1), 22–30. PMC6372966 30630306

[B5] Blevins A. M. Lashinsky J. N. McCammon C. Kollef M. Micek S. Juang P. (2019). Incidence of acute kidney injury in critically ill patients receiving vancomycin with concomitant piperacillin-tazobactam, cefepime, or meropenem. Antimicrob. Agents Chemother. 64 (5), 026588-18–e2719. 10.1128/AAC.02658-18 PMC6496064 30782987

[B6] Chinese Society of Cardiology (2014). Expert consensus on prevention, diagnosis, and treatment of infective endocarditis in adults. Chin. J. Cardiovasc. Dis. 42 (10), 806–816. 10.3760/cma.j.issn.0253-3758.2014.10.004 25547443

[B7] Choi Y. C. S. S. Soliman D. Bingham A. L. Pontiggia L. Hunter K. (2017). Intravenous vancomycin associated with the development of nephrotoxicity in patients with class III obesity. Ann. Pharmacother. 51 (11), 937–944. 10.1177/1060028017720946 28709394

[B8] Chun J. Y. Song K. H. Lee D. E. Hwang J. H. Jung H. G. Heo E. (2021). Impact of a computerised clinical decision support system on vancomycin loading and the risk of nephrotoxicity. Int. J. Med. Inf. 149, 104403. 10.1016/j.ijmedinf.2021.104403 33592353

[B9] Contejean A. Tisseyre M. Canoui E. Treluyer J. M. Kerneis S. Chouchana L. (2021). Combination of vancomycin plus piperacillin and risk of acute kidney injury: a worldwide pharmacovigilance database analysis. J. Antimicrob. Chemother. 76 (5), 1311–1314. 10.1093/jac/dkab003 33617641

[B10] Cosgrove S. E. Vigliani G. A. Fowler V. G. Abrutyn E. Corey G. R. Levine D. P. (2009). Initial low-dose gentamicin for *Staphylococcus aureus* bacteremia and endocarditis is nephrotoxic. Clin. Infect. Dis. 48 (6), 713–721. 10.1086/597031 19207079

[B11] Elyasi S. Khalili H. Dashti-Khavidaki S. Mohammadpour A. (2012). Vancomycin-induced nephrotoxicity: mechanism, incidence, risk factors and special populations. A literature review. Eur. J. Clin. Pharmacol. 68 (9), 1243–1255. 10.1007/s00228-012-1259-9 22411630

[B12] Filippone E. J. Kraft W. K. Farber J. L. (2017). The nephrotoxicity of vancomycin. Clin. Pharmacol. Ther. 102 (3), 459–469. 10.1002/cpt.726 28474732 PMC5579760

[B13] Gagneux-Brunon A. Pouvaret A. Maillard N. Berthelot P. Lutz M. F. Cazorla C. (2019). Acute kidney injury in infective endocarditis: a retrospective analysis. Med. Mal. Infect. 49 (7), 527–533. 10.1016/j.medmal.2019.03.015 30955847

[B14] Goenaga Sanchez M. A. Kortajarena Urkola X. Bouza Santiago E. Muñoz García P. Verde Moreno E. Fariñas Álvarez M. C. (2017). Aetiology of renal failure in patients with infective endocarditis. The role of antibiotics. Med. Clin. Barc. 149 (8), 331–338. 10.1016/j.medcli.2017.03.009 28431897

[B15] Habib G. Lancellotti P. Antunes M. J. Bongiorni M. G. Casalta J. P. Del Zotti F. (2015). 2015 ESC guidelines for the management of infective endocarditis: the task force for the management of infective endocarditis of the European society of Cardiology (ESC). Endorsed by: European association for cardio-thoracic surgery (EACTS), the European association of nuclear medicine (EANM). Eur. Heart J. 36 (44), 3075–3128. 10.1093/eurheartj/ehv319 26320109

[B16] He N. Su S. Ye Z. Du G. (2020). Evidence-based guideline for therapeutic drug monitoring of vancomycin: 2020 update by the division of therapeutic drug monitoring, Chinese pharmacological society. Clin. Infect. Dis. 71 (Suppl. 4), S363–S371. 10.1093/cid/ciaa1536 33367582

[B17] Huang W. C. Wang M. T. Lai T. S. Lee K. H. Shao S. C. Chen C. H. (2022). Nephrotoxins and acute kidney injury - the consensus of the Taiwan acute kidney injury Task Force. J. Formos. Med. Assoc. 121 (5), 886–895. 10.1016/j.jfma.2021.12.007 34998658

[B18] Jeffres M. N. (2017). The whole price of vancomycin: toxicities, troughs, and time. Drugs 77 (11), 1143–1154. 10.1007/s40265-017-0764-7 28573434 PMC5501899

[B19] Khwaja A. (2012). KDIGO clinical practice guidelines for acute kidney injury. Nephron Clin. Pract. 120 (4), c179–c184. 10.1159/000339789 22890468

[B20] Kiley P. S. P. A. Hodge L. A. Kaplan M. C. Baczek S. M. Stanley J. S. (2022). Retrospective cohort study of the incidence of acute kidney injury with vancomycin area under the curve-based dosing with concomitant piperacillin-tazobactam compared to meropenem or cefepime. Antimicrob. Agents Chemother. 66 (8), e0004022. 10.1128/aac.00040-22 35867523 PMC9380555

[B21] Kim J. Y. Kim K. Y. Yee J. Gwak H. S. (2022). Risk scoring system for vancomycin-associated acute kidney injury. Front. Pharmacol. 13, 815188. 10.3389/fphar.2022.815188 35330832 PMC8940364

[B22] Kunming P. Can C. Zhangzhang C. Wei W. Qing X. Xiaoqiang D. (2021). Vancomycin associated acute kidney injury: a longitudinal study in China. Front. Pharmacol. 12, 632107. 10.3389/fphar.2021.632107 33762952 PMC7982802

[B23] Kunming P. Xiaotian J. Qing X. Chenqi X. Xiaoqiang D. Qian Zhou L. (2023). Impact of pharmacist intervention in reducing vancomycin-associated acute kidney injury: a systematic review and meta-analysis. Br. J. Clin. Pharmacol. 89 (2), 526–535. 10.1111/bcp.15301 35285970

[B24] Legrand M. Pirracchio R. Rosa Petersen M. L. Van der Laan M. Fabiani J. N. (2013). Incidence, risk factors and prediction of post-operative acute kidney injury following cardiac surgery for active infective endocarditis: an observational study. Crit. care 17 (5), R220. 10.1186/cc13041 24093498 PMC4056899

[B25] Liu C. Yan S. Wang Y. Wang J. Fu X. Song H. (2021). Drug-induced hospital-acquired acute kidney injury in China: a multicenter cross-sectional survey. Kidney Dis. (Basel). 7 (2), 143–155. 10.1159/000510455 33824870 PMC8010232

[B26] Mehran R. Dangas G. D. Weisbord S. D. (2019). Contrast-associated acute kidney injury. N. Engl. J. Med. 380 (22), 2146–2155. 10.1056/NEJMra1805256 31141635

[B27] Muklewicz J. D. Steuber T. D. Edwards J. D. (2021). Evaluation of area under the concentration-time curve-guided vancomycin dosing with or without piperacillin-tazobactam on the incidence of acute kidney injury. Int. J. Antimicrob. Agents 57 (1), 106234. 10.1016/j.ijantimicag.2020.106234 33232734

[B28] Nakatani S. Ohara T. Ashihara K. Izumi C. Iwanaga S. Eishi K. (2019). JCS 2017 guideline on prevention and treatment of infective endocarditis. Circ. J. 83 (8), 1767–1809. 10.1253/circj.CJ-19-0549 31281136

[B29] O'Callaghan K. Hay K. Lavana J. McNamara J. F. (2020). Acute kidney injury with combination vancomycin and piperacillin-tazobactam therapy in the ICU: a retrospective cohort study. Int. J. Antimicrob. Agents 56 (1), 106010. 10.1016/j.ijantimicag.2020.106010 32413387

[B30] Pan K. Ma L. Xiang Q. Li X. Li H. Zhou Y. (2017). Vancomycin-associated acute kidney injury: a cross-sectional study from a single center in China. PLoS One 12 (4), e0175688. 10.1371/journal.pone.0175688 28426688 PMC5398886

[B31] Pan K. M. Wu Y. Chen C. Chen Z. Z. Xu J. A. Cao L. (2018). Vancomycin-induced acute kidney injury in elderly Chinese patients: a single-centre cross-sectional study. Br. J. Clin. Pharmacol. 84 (8), 1706–1718. 10.1111/bcp.13594 29607531 PMC6046479

[B32] Ritchie B. M. Hirning B. A. Stevens C. A. Cohen S. A. DeGrado J. R. (2017). Risk factors for acute kidney injury associated with the treatment of bacterial endocarditis at a tertiary academic medical center. J. Chemother. 29 (5), 292–298. 10.1080/1120009X.2017.1296916 28245728

[B33] Rybak M. Lomaestro B. Rotschafer J. C. Moellering R. Craig W. Billeter M. (2009). Therapeutic monitoring of vancomycin in adult patients: a consensus review of the American society of health-system pharmacists, the infectious diseases society of America, and the society of infectious diseases pharmacists. Am. J. Health Syst. Pharm. 66 (1), 82–98. 10.2146/ajhp080434 19106348

[B34] Rybak M. J. Le J. Lodise T. P. Levine D. P. Bradley J. S. Liu C. (2020). Therapeutic monitoring of vancomycin for serious methicillin-resistant *Staphylococcus aureus* infections: a revised consensus guideline and review by the American society of health-system pharmacists, the infectious diseases society of America, the pediatric infectious diseases society, and the society of infectious diseases pharmacists. Am. J. Health Syst. Pharm. 77 (11), 835–864. 10.1093/ajhp/zxaa036 32191793

[B35] von Elm E. Altman D. G. Egger M. Pocock S. J. Gøtzsche P. C. Vandenbroucke J. P. (2007). The Strengthening the Reporting of Observational Studies in Epidemiology (STROBE) statement: guidelines for reporting observational studies. Lancet 370 (9596), 1453–1457. 10.1016/S0140-6736(07)61602-X 18064739

[B36] Yang L. Xing G. Wang L. Wu Y. Li S. Xu G. (2015). Acute kidney injury in China: a cross-sectional survey. Lancet 386 (10002), 1465–1471. 10.1016/S0140-6736(15)00344-X 26466051

