# Supplementary material for: Vancomycin associated acute kidney injury in patients with infectious endocarditis: a large retrospective cohort study
Source: Front Pharmacol. 2023 Nov 13;14:1260802. doi: 10.3389/fphar.2023.1260802 (PMC10679345; doi:10.3389/fphar.2023.1260802)
Supplement: Supplementary file 1 [file Table1.docx]

Supplementary Table 1 Demographic information, clinical characteristics, vancomycin exposure and concomitant nephrotoxic drugs of VA-AKI patients with unrecovered or recovered kidney function

| Factors | Total  N = 74 | Patients with unrecovered kidney function N = 24 | Patients with recovered kidney function N = 54 | P Value |
| --- | --- | --- | --- | --- |
| Demographic information | | | | |
| Gender (male) | 53 (71.6) | 15 (75.0) | 38 (70.4) | 0.70 |
| Age (years) | 58.0 (22) | 62.0 (16.0) | 56.5 (21.0) | 0.041 |
| Body Mass Index (Kg/m^2^)* | 22.0 (3.8) | 22.0 (3.2) | 22.0 (4.0) | 0.87 |
| Payment mode (At one's own expense) | 52 (70.2) | 9 (45.0) | 43 (79.6) | 0.004 |
| Baseline serum creatinine μmol/L | 86.3 (41.0) | 91.5 (47.7) | 84.5 (35.0) | 0.27 |
| Concomitant underlying diseases | | | | |
| Coronary heart disease | 3 (4.0) | 2 (10.0) | 1 (1.9) | 0.18^f^ |
| Hypertension | 13 (17.5) | 6 (30.0) | 7 (13.0) | 0.17^b^ |
| Diabetes | 7 (9.4) | 3 (15.0) | 4 (7.4) | 0.59^b^ |
| Heart failure | 11 (14.8) | 4 (20.0) | 7 (13.0) | 0.70^b^ |
| Sepsis | 25 (33.7) | 6 (30.0) | 19 (35.2) | 0.68 |
| Cancer | 7 (9.4) | 3 (15.0) | 4 (7.4) | 0.59^b^ |
| Valvular heart disease | 67 (90.5) | 16 (80.0) | 51 (94.4) | 0.15^b^ |
| Kidney insufficiency | 3 (4.0) | 2 (10.0) | 1 (1.9) | 0.18^f^ |
| Severity of illness | | | | |
| Cardiac surgery | 68 (91.8) | 15 (75.0) | 53 (98.1) | 0.006^b^ |
| Admission to the ICU | 45 (60.8) | 9 (45.0) | 36 (66.7) | 0.09 |
| Mechanical ventilation | 14 (18.9) | 5 (25.0) | 9 (16.7) | 0.63^b^ |
| Length of stay | 18.0 (12.0) | 18.6 (13.1) | 17.5 (13.2) | 0.36 |
| Vancomycin exposure |  |  |  |  |
| Vancomycin varieties |  |  |  | 0.40 |
| Wen Kexin | 50 (675.) | 12 (60.0) | 38 (70.4) |  |
| Lai Kexin | 24 (32.4) | 8 (40.0) | 16 (29.6) |  |
| Receiving TDM | 41 (55.4) | 8 (40.0) | 33 (61.1) | 0.087 |
| Length of vancomycin therapy mean (median), range | 12.0 (10.6), 1.5-49.5 | 11.0 (8.9),  2.5-32.5 | 12.8 (11.1),  1.5-49.5 | 0.46 |
| Daily dose |  |  |  | 0.63^b^ |
| ≤2 g/d | 70 (94.5) | 18 (90.0) | 52 (96.3) |  |
| > 2 g/d | 4 (5.4) | 2 (10.0) | 2 (3.7) |  |
| Concomitant nephrotoxic drugs | 67 (90.5) | 18 (90.0) | 49 (90.7) | 1.00^b^ |
| Vasopressors | 22 (29.7) | 2 (10.0) | 20 (37.0) | 0.024 |
| Loop diuretics | 63 (85.1) | 18 (90.0) | 45 (83.3) | 0.73^b^ |
| Aminoglycosides | 1 (1.3) | 1 (5.0) | 0 | 0.27^f^ |
| Cephalosporins | 18 (24.3) | 7 (35.0) | 11 (20.4) | 0.32^b^ |
| Carbapenems | 40 (54.0) | 7 (35.0) | 33 (61.1) | 0.045 |
| RAS blockers | 10 (13.5) | 4 (20.0) | 6 (11.1) | 0.35^b^ |
| Radiocontrast agents | 17 (22.9) | 6 (30.0) | 17 (31.5) | 0.90 |
| NSAIDs | 4 (5.4) | 1 (5.0) | 3 (5.6) | 1.00^b^ |
| Glutathione | 13 (17.5) | 5 (25.0) | 8 (14.8) | 0.50^b^ |
| Coenzyme Q10 | 9 (12.1) | 2 (10.0) | 7 (13.0) | 1.000^b^ |

Data are described as mean (SD), n (%), or median (IQR). b refers to the calibration of the chi-square test. ICU= intensive care unit. NSAIDs= Non-steroidal anti-inflammatory drugs. VA-AKI= vancomycin-associated kidney injury. RAS blockers = Renin-angiotensin system blockers. TDM= therapeutic drug monitoring

*There were missing values in the Body Mass Index data. The percentage of missing values for total was 8.1% (6) [5.5% (3) for patients with recovered kidney function; 15.0% (3) for patients with unrecovered kidney function]. Missing values were filled using the median.
